# Sediment properties modulate the bioturbation effects of *Tubifex* spp. on CH_4_ and CO_2_ emissions in freshwater ecosystems

**DOI:** 10.1007/s10533-026-01364-6

**Published:** 2026-08-19

**Authors:** Ilenia Marquina-Luevano, Lucía Cabrera-Lamanna, Annelies J. Veraart, Andreas Lorke, Dick van Oevelen, Sarian Kosten

**Affiliations:** 1 Department of Ecology, Radboud Institute for Biological and Environmental Sciences, Radboud University, Nijmegen, the Netherlands; 2 Institute for Environmental Sciences, University of Kaiserslautern-Landau (RPTU), Landau, Germany; 3 Department of Estuarine and Delta Systems, Royal Netherlands Institute for Sea Research – NIOZ, Yerseke, the Netherlands

**Keywords:** Bioturbation, *Tubifex*, Greenhouse gases, Sediment properties, Freshwater ecosystems

## Abstract

Freshwater ecosystems are major natural sources of methane (CH_4_) and carbon dioxide (CO_2_), with large amounts of these gases being produced in sediments. Bioturbators such as *Tubifex* spp. have been proposed to alter the dynamics of both gases as they inhabit the sediment–water interface, influencing biogeochemical processes. Although eutrophication generally leads to strong shifts in the benthic community, *Tubifex* spp. tolerate eutrophication. However, their role in modulating greenhouse gas (GHG) emissions requires further elucidation. We used 24 microcosm to assess the presence of *Tubifex* spp. on diffusive and ebullitive CH_4_ and CO_2_ emissions across three eutrophic sediments: one with clay (C) and two with peat, containing low and high organic matter content (P-lom and P-hom, respectively). The effects of *Tubifex* on carbon emissions varied across emission pathways and were modulated by sediment types. *Tubifex* presence tended to alter diffusive CH_4_ and CO_2_ fluxes, leading to higher mean CO_2_ and total mean CH_4_ emissions in P-lom sediments, whereas weak effects of bioturbation were observed in P-hom and C sediments. The variation in effects was likely associated with sediment-dependent differences in *Tubifex* bioturbation intensity, as well as Tubificid-mediated shifts in interactions among organic carbon dynamics, CH_4_-cycling microbial abundances, nutrient availability and gaseous carbon fluxes. We did not find evidence that *Tubifex* affected ebullitive CH_4_ emissions, sediment oxygenation or the abundance of methanogens and methanotrophs in the sediment. We conclude that to incorporate their effects in GHG emission estimates, more context-dependent insights are needed.

**Supplementary Information:**

The online version contains supplementary material available at https://doi.org/10.1007/s10533-026-01364-6.

## Introduction

Freshwater ecosystems are the main natural sources of methane (CH_4_) and important sources of carbon dioxide (CO_2_), accounting for 41% and 15% of global CH_4_ and CO_2_ emissions, respectively (Bastviken et al. 2011; Cole et al. 2007; Holgerson & Raymond 2016). Under anoxic conditions, methanogenic archaea (methanogens) degrade organic matter (OM) and produce CH_4_ as an end product. The CH_4_ production rate depends on the availability of terminal electron acceptors (TEAs), OM availability and composition, and temperature (Gudasz et al. 2010; Tranvik et al. 2009). Especially under aerobic conditions, a large share of the CH_4_ produced is oxidized to CO_2_ by methane-oxidizing bacteria (MOB); thus, CH_4_ emissions to the atmosphere are eventually the net difference between production and oxidation (Bastviken et al. 2008; Borrel et al. 2011). While microbial oxidation can decrease dissolved CH_4_ concentrations in the upper layers of the sediment and in the water column, CH_4_ released via ebullition largely escapes oxidation and can contribute significantly to total emissions (Aben et al. 2017; Bastviken et al. 2008, 2011). In contrast, CO_2_ production depends mostly on OM mineralization by aerobic microorganisms (Cole et al. 2007; Duarte & Prairie 2005). Because the availability of TEAs and OM is of paramount importance for the production and consumption rates of CH_4_ and CO_2_, it is not surprising that bioturbators—known for their ability to redistribute dissolved and particulate OM as well as oxygen (O_2_) and dissolved nutrients— (Bartoli et al. 2020; Chakraborty et al. 2022; Chen et al. 2016; Kristensen et al. 2012; Nogaro et al. 2009) have been suggested to impact CH_4_ and CO_2_ emissions (Benelli & Bartoli 2021; Bonaglia et al. 2017; Booth et al. 2021).

*Tubifex* species are among the most abundant and widespread bioturbators of lentic freshwater systems and are widely found in the Netherlands (Nijboer et al. 2004). As they feed head-down in the sediment, they are classified as upward-conveyors, creating burrow galleries that enhance sediment–water exchanges, vertical material transport and sediment reworking (Hietanen et al. 2007; Lagauzère et al. 2009; Nogaro et al. 2009). These processes are driven primarily by their downward movement to feed on OM in deeper sediment layers and the ejection of faecal pellets at the surface (Chakraborty et al. 2022; Kristensen et al. 2012). Their high tolerance to hypoxia enables them to outcompete other benthic organisms, which is particularly favourable in eutrophic environments (Fiskal et al. 2020). The projected global increase in eutrophication of inland waters (Meerhoff et al. 2022) could lead to a potential expansion of suitable *Tubifex* habitats. While eutrophication is known to enhance CH_4_ emissions mainly due to increased availability of OM—causing anoxia and increasing CH_4_ production (Beaulieu et al. 2019)—simultaneous increases in CH_4_ oxidation related to bioturbation-induced processes may partially offset the production. However, this mechanism remains unclear as studies examining the effects of *Tubifex* spp. on carbon emissions are limited and present contrasting results (Colina et al. 2021; Mehring et al. 2017; Temmink et al. 2021).

*Tubifex* spp. may influence CH_4_ and CO_2_ emissions through bioturbation-driven physicochemical and biological changes at the sediment–water interface, with their mixing activities extending to depths up to 10 cm (Lagauzère et al. 2009; Rodriguez et al. 2001). The physical movement of *Tubifex* enhances oxygen penetration into sediments, potentially stimulating aerobic microbial activity in the sediment (Mermillod-Blondin et al. 2001; Navel et al. 2012; Yang et al. 2020). *Tubifex* can also reduce O_2_ availability in the sediment, due to their own respiration, thereby promoting anaerobic microbial processes (Boeker et al. 2016; Mermillod‐Blondin et al. 2004). These contrasting findings, along with the lack of a clear process-based understanding of Tubificid-mediated effects on sediment biogeochemistry, highlight the need for studies on Tubificid-induced changes in MOB and methanogen communities. Depending on the strength of the contrasting impacts on CH_4_ production and oxidation, sediment–water diffusive CH_4_ emissions may either increase or decrease in the presence of *Tubifex*, along with a potential increase in CO_2_ emissions.

The formation of CH_4_ bubbles and their subsequent release via ebullition strongly depend on factors that alter not only CH_4_ production rates but also the hydrostatic pressure and the effective diffusion coefficient in the sediment (Bastviken et al. 2004; Langenegger et al. 2019). *Tubifex* burrowing enhances the effective diffusion coefficient through alterations in sediment structure, porosity, and water-holding capacity (Navel et al. 2012; Nogaro et al. 2009), potentially increasing sediment–water CH_4_ fluxes (Sweerts et al. 1991). Increased diffusion reduces the build-up of CH_4_ in the sediment porewater, which may reduce CH_4_ bubble formation in sediments (Booth et al. 2021). Because the interaction between *Tubifex* spp. and sediment properties is bidirectional —*Tubifex* can alter sediment structure, and sediment structure can modulate *Tubifex’s* bioturbation activities and effects (Mermillod-Blondin 2011)— it is crucial to understand how *Tubifex* impact carbon emissions under different sediment contexts.

**Fig. 1:**
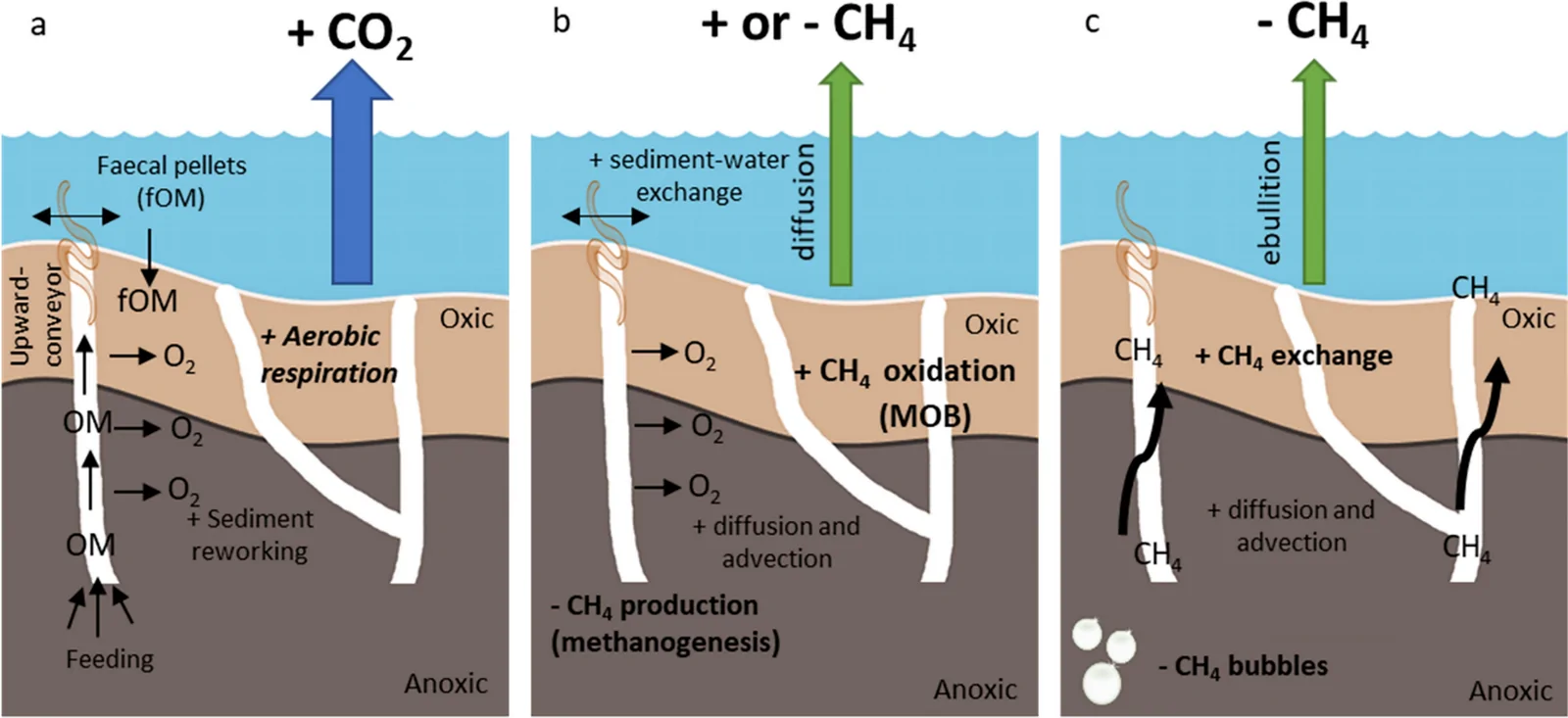
Hypothesized effects of *Tubifex* spp. on methane (CH_4_) and CO_2_ emissions in shallow lentic systems. **a** Effects on diffusive CO_2_ emissions, **b** Effects on diffusive CH_4_ emissions and **c** Effects on ebullitive CH_4_ emissions. “ + ” = increased, “- ‘” = decreased, “fOM” = fresh and labile organic matter, “OM” = organic matter, “MOB” = methane-oxidizing bacteria

In this study, we aim to test the effects of *Tubifex* spp. on CH_4_ and CO_2_ greenhouse gas (GHG) emissions from microcosms mimicking shallow lentic systems that differ in sediment composition (i.e., OM percentage, DOC concentrations and soil type). We hypothesize that *Tubifex* spp. bioturbating activity increases CO_2_ emissions by mobilising labile OM, and enhancing aerobic decomposition (sediment reworking, Fig. 1a). Methane emissions may either decrease or increase depending of the net effect of increased CH_4_ sediment–water transport and increased CH_4_ oxidation related to an increase in O_2_ penetration in sediments, in turn increasing the abundance of methanotrophs while potentially also decreasing that of methanogens (Fig. 1b). In addition, the extensive burrow galleries promoting the higher CH_4_ exchange from sediment porewater to surface water, decrease CH_4_ bubble formation (ebullition, Fig. 1c).

To test our hypotheses, we measured total CH_4_ (i.e. diffusive + ebullitive) and CO_2_ emissions in a controlled laboratory experiment using microcosms with and without *Tubifex* spp. during 62 days. Additionally, we analysed the following parameters: (1) sediment reworking depth, (2) O_2_ concentrations, (3) methanogenic and MOB abundance and (4) nutrient and CH_4_ concentrations in surface and porewater to unravel the physicochemical and biological controls of *Tubifex* spp. on carbon emissions.

## Materials and methods

### Sediment collection

Three different sediments were collected from eutrophic ditches in the Netherlands. The selected sites were located in an area that features clay soils (51°54′35.6"N 5°46′47.7" E) located in Valburg (C) draining a dairy farm, peat soils (53.0047° N, 5.9608° E) located in Gersloot (P-lom) near farmland, and peat soils (52°06′41.8"N 4°50′39.1"E) located in Zegveld (P-hom) near a dairy farm. These sediments differed in OM, water and DOC content (Table 1). After collection, all sediments were sieved through a 250 µm mesh to remove macrofauna and debris. Sieved sediments were kept for around 6 days at 4 °C until setting up the microcosms. Before filling the microcosms, we used a mechanical stirring machine to homogenise the sediment.

**Table 1:** Overview of sediment characteristics at the beginning of the experiment

Parameters	C	P-lom	P-hom
OM	7%	49%	65%
DOC-pw	23.43 mg L^−1^	73.68 mg L^−1^	27.73 mg L^−1^
Water content	50%	87%	84%
Location soil type	Clay	Peat	Peat
Location name	Valburg	Gersloot	Zegveld

*OM* organic matter, *DOC-pw* dissolved organic carbon in porewater, *C* Clay, *P-lom* peat with low *OM and P-hom* = Peat with high OM.

### Tubifex origin and pre-treatment

Tubificid worms were obtained from a commercial aquarium store (De Maanvis, Nijmegen, the Netherlands). To achieve our desired *Tubifex* spp. density, we weighed five sets of 20 Tubificid worms. Each total weight was then divided by 20 and averaged to calculate the approximate biomass weight of one individual. Around 1248 individuals (~ 4.89 g) were introduced per microcosm to reach an initial density of 30,000 ind./m^2^ mimicking densities previously reported in natural and seminatural aquatic ecosystems (Mermillod‐Blondin et al. 2018; Nogaro et al. 2009). *Tubifex* spp. added to the sediment had an acclimation period of 14 days, after this time, we started the experimental measurements. After 62 days (counting from the date *Tubifex* spp. were added), at the end of the experiment (62 days after *Tubifex* sp. addition), the worms were counted again (see Supplementary Information [SI], Section M4 for details).

### Experimental setup

Our experimental design consisted of twenty-four microcosms (0.32 m height, 0.32 m width and 0.13 m depth, ~ 13.3 L) containing 5.4 L of sediment (15 cm height) from one of each location and 4 L of artificial rainwater (10 cm height; SI, Table S1). This resulted in eight microcosms filled with each sediment, from which half received *Tubifex* spp. and the others did not (Control). On top of the sediment of each microcosm a 0.5 cm slice of a mixture of luminophores (80–180 μm), which are fluorescent-dyed sediment particles that are used in experiments with macrofauna to measure various sediment-mixing parameters, as described in Fernandes et al., (2006), and artificial sediment (1:20) enriched with “fresh” organic matter (SI, Table S2) was placed to examine sediment reworking depth. A 5 mm thick layer of this mixture was frozen for 24 h at −20 °C. The frozen material was cut into the shape of the microcosms and placed on top of the sediments to enable equal distribution (SI, Fig. S1). Adding a luminophore layer on the surface sediment ensured that the initial tracer depth was defined once it melted (i.e., at the very surface: 0 cm).

All microcosms were randomly placed in a climatized room at a constant air temperature of 20 °C under dark conditions for 62 days. To prevent the formation of surface microfilms that could alter gas exchange, we implemented a low-flow system by continuously and slowly dripping aerated artificial rainwater into the microcosms (for details see SI, section M3). This flow-through setup ensured gentle water movement while minimizing the effect on GHG exchange and introducing a small amount of O_2_ to avoid complete anoxia. Despite the addition of aerated water, the microcosms were consistently characterized by low, non-saturated O_2_ conditions throughout the whole experimental period, which are typical of eutrophic freshwater systems.

### Diffusive greenhouse gas (GHG) flux measurements

Diffusive GHG measurements were conducted every 7 days. Methane and CO_2_ diffusive emissions were measured in the headspace of each microcosm using an acrylic rectangular lid (0.32 × 0.13 m) with a rubber seal ensuring a gastight connection between the lid and the microcosm (SI, Fig.S1-b). The lid had two connection ports that were connected to the inflow and outflow of a Picarro G2508 Greenhouse Gas Analyzer (Picarro Inc., Santa Clara, CA, USA). Measurements were conducted until a linear increase of CH_4_ and CO_2_ was observed for 1–5 min. The diffusive emissions were calculated based on the slope between gas concentration and time, as in Almeida et al. (2016). Sudden increases in CH_4_ concentrations (presumably due to ebullition) were discarded from the diffusive emission calculations.

### Methane ebullition

The ebullitive emissions were calculated by measuring bubble release and the CH_4_ concentration in the emitted bubbles. Bubble release was measured using bubble traps, which consisted of an inverted plastic funnel (Ø 0.065 m) connected to a water-filled glass exetainer (12 mL, Labco Limited, High Wycombe, UK) from which we had removed the bottom. The gas volume accumulated in the traps was collected every 3–4 days with a syringe through the rubber septum in the lid of the exetainer. Every second time we collected gas, a 3 mL sub-sample was stored inside a 3 mL vacuum Exetainer (Labco Limited, High Wycombe, UK) at ambient temperature for later analysis of the CH_4_ concentration.

Methane concentrations were measured by injecting 100 µl of gas on a HP 5890 GC gas chromatograph equipped with flame ionization detection using a Porapak Q column (80/200 mesh, Avondale, CA, USA). This was done in triplicate.

### Water physicochemical measurements

To analyse nutrient concentrations of phosphate (PO_4_^3−^), ammonium (NH_4_^+^) and nitrate (NO_3_^−^) in surface water, we collected samples at 5 cm below the water surface with a 60 mL syringe. Surface water was immediately filtered using a conical vacuum flask and GF/C Whatman filters. For porewater water extraction, we connected a syringe to a permanently placed dialysis porewater sampler (pore size 0.12/0.18 µm, Rhizon SMS 10 cm, Wageningen, the Netherlands). All samples were stored in 10 mL plastic containers at −20 ˚C until further analysis. The concentrations of PO_4_^3−^, NH_4_^+^ and NO_3_^−^ were measured on an autoanalyzer using colorimetric methods (III, Seal Analytical, Norderstedt, Germany). Filtered surface and porewater were used to measure DOC and DN concentrations on a total organic carbon analyser (TOC-L CPH/CPN, Shimadzu). Total inorganic carbon (TIC) was measured on an ABB Advance optima Infrared Gas Analyzer (IRGA, Frankfurt, Germany) by injecting unfiltered (surface) and filtered (pore) water samples inside an N_2_ flushed chamber containing 1 ml 0.4 M H_3_PO_4_. All water samples were taken at the beginning and end of our experiment.

The dissolved O_2_ concentrations (mg/L) in the surface water of each microcosm were measured every 14 days at 5 cm below the water surface (SI, Fig. S1-c), using a Portable Multi Meter (HQ2200, HACH, Loveland, CO, USA) with the attached probe (LDO1010). Sediment oxygenation was measured at the end of the experiment as explained in SI, section M6.

### Sediment reworking depth

To quantify sediment reworking depth (bioturbation) at the end of the experiment, two vertical sediment cores (Ø 2.5 cm) were collected from each microcosm and stored at − 20 °C for three weeks before analysis. Frozen cores were sliced at 0.5 cm intervals, and luminophore particles were quantified via image analysis. High-resolution images were taken of each slice using a Canon EOS-1300D (5184 × 3456 pixels) under full UV illumination (Ayra UV LED Brick blacklight-effect). Image analysis was performed in ImageJ (v2.14.0) using a custom macro that applied a standardized black:white threshold to distinguish pink luminophore particles from background sediment. For more details, see SI, section M7. To account for slight variations in core alignment and sediment layers, the counting area was manually adjusted using the “SetTool” (“oval”) function, excluding core edges (SI, Fig. S2).

The number of luminophores found in each sediment layer were counted visually. Subsequently, we summed all luminophores transported into the sediment profile (ΣLUM) and estimated the maximum sediment reworking depth (MSRD). The ΣLUM was used to assess the ability of bioturbators to transfer particles from the sediment–water interface to deeper sediment layers (Trannum 2017). The MSRD was defined as the depth where 95% of the total luminophore particles were counted (Lagauzère et al. 2009) and was calculated using linear interpolation.

### Sediment analyses

At the end of the experiment, sediment samples from the upper 1 cm (methanotrophs) and 5 cm (methanogens) depth from each microcosm were extracted using pre-cut 10 ml sterile syringes and immediately stored at −20 °C until further molecular analysis. Organic matter content was determined as loss on ignition (70 °C drying followed by combustion at 550 °C; Heiri et al. 2001). Sediment carbon (C) and nitrogen (N) contents were measured on an elemental analyzer (Vario Micro Cube, Elementar, Langenselbold, Germany).

Methanogens and methane-oxidizing bacteria (MOB) abundance.

The DNA was extracted from 0–5 cm (for methanogens) and 0–1 cm (for MOB) sediment layers using the DNeasy PowerSoil Pro kit (Qiagen, Venlo, the Netherlands), with some small modifications to improve DNA yield from relatively wet sediments (Nijman et al. 2021). After extraction, we measured and diluted the DNA to achieve concentrations of 0.25 µg/µl (+—0.15) using a Qubit fluorometer (Invitrogen). Functional marker genes *pmoA* (particulate methane monooxygenase) and *mcrA* (encoding for methyl coenzyme-M reductase) were used as functional marker genes of MOB and methanogens, respectively (Grabarse et al. 2000; Stolyar et al. 1999), and measured by quantitative polymerase chain reaction (qPCR), on a BioRad iQ5 Multicolour Real-Time PCR detection system (Vers. 2.0, BioRad, Gothenburg, Sweden), using primer combinations A189F and Mmb661R for *pmoA* (Costello & Lidstrom 1999) and mlas and mcrA-rev for *mcrA* (Steinberg & Regan 2008), see SI, Table S3 and section M8, for sequences, qPCR programmes and reagent concentrations. To quantify gene abundances, we prepared a standard curve using tenfold serial dilutions of cDNA with six known plasmid concentrations (1.25—1.25 × 10^–7^ copy numbers) expressing our genes of interest. All samples and standards were measured in triplicate.

### ***Stable carbon isotope ratios δ***^***13***^***C-Macrofauna and sediment***

To assess potential carbon sources for *Tubifex* spp., δ^13^C signatures of sediment and macrofauna were analyzed at the end of the experiment. *Tubifex* individuals and sediment samples were dried at 70 °C (7 days and ≥ 72 h, respectively), homogenized, weighed into tin capsules, and analyzed using a Flash 2000 elemental analyzer coupled to a Delta V Advantage isotope ratio mass spectrometer (IRMS, Thermo Scientific, Cumbria, UK).

### Data analysis

All statistical analyses were carried out using R version 4.4.1. Linear-mixed effects models (LMMs) fitted with replicates as a random factor and sediment, treatment and time as fixed factors were conducted using the package “*lme4*” (Bates et al. 2015) to evaluate differences between *Tubifex* spp. and control treatments on CH_4_ and CO_2_ emissions during the whole experimental period. Model assumptions, including linearity, homoscedasticity, and normality, were evaluated using residual plots, histograms, Q-Q plots of residuals, and the Shapiro–Wilk test. The significance of fixed effects was determined via Type III ANOVA, with degrees of freedom estimated using the Kenward-Roger approximation. Additionally, we calculated the mean emissions of CH_4_ (diffusive, ebullitive and total = diffusive + ebullitive) and CO_2_ as explained in SI, section M9. We used linear models (LMs) to test the differences between *Tubifex* spp. and control treatments on mean CH_4_ and CO_2_ using the package “*car*” (Fox & Weisberg 2019).

To assess the effects of treatment, sediment type, and their interaction on microbial abundance and sediment reworking, a two-way analysis of variance (ANOVA) or an LM was performed using the package “*car*”. For all LMs, LMMs and ANOVAs, pairwise comparisons of treatment effects were performed within each sediment type using the “*emmeans”* package (Lenth 2025) and adjusted using Tukey’s method.

Three Partial Least Squares (PLS) were performed using the “*pls*” package (Liland et al. 2024) to assess which benthic and pelagic variables (*X* variables) best explained mean CH_4_ (diffusion and ebullition) and CO_2_ emissions (*Y* variables) in ‘control’ and ‘*Tubifex* spp.’ microcosms. All analyses included the following independent benthic variables: NO_3_^−^, PO_4_^3−^, NH_4_^+^, DOC, OM, TIC, *mcrA*, *pmoA*, *Tubifex* density and δ^13^C of the sediment, and independent pelagic variables: DOC, O_2_ and TIC. We used scaled and centred PLS coefficients to interpret results. The ranking of importance of *X* variables explaining *Y* variables was measured by extracting their Variable Importance Projection (VIP). Variables with a VIP greater than 1 were considered highly influential, while those between 0.8 and 1.0 were considered moderately important. Finally, variables with VIP scores below 0.8 were considered to have low influence.

Before all analyses, highly skewed data (non-normal) were log-transformed to achieve normality of residuals, while figures display the untransformed data. For the sediment reworking depth (MSDR%) analysis, one microcosm was excluded due to a methodological error when placing the luminophores. Four core replicates were also discarded as they defrosted before being able to capture an image. After excluding ebullition interference, one sampling date for diffusive emissions was excluded from analysis due to the absence of a comparison between treatments.

## Results

### *Tubifex* effects on methane and carbon dioxide emissions

**Fig. 2:**
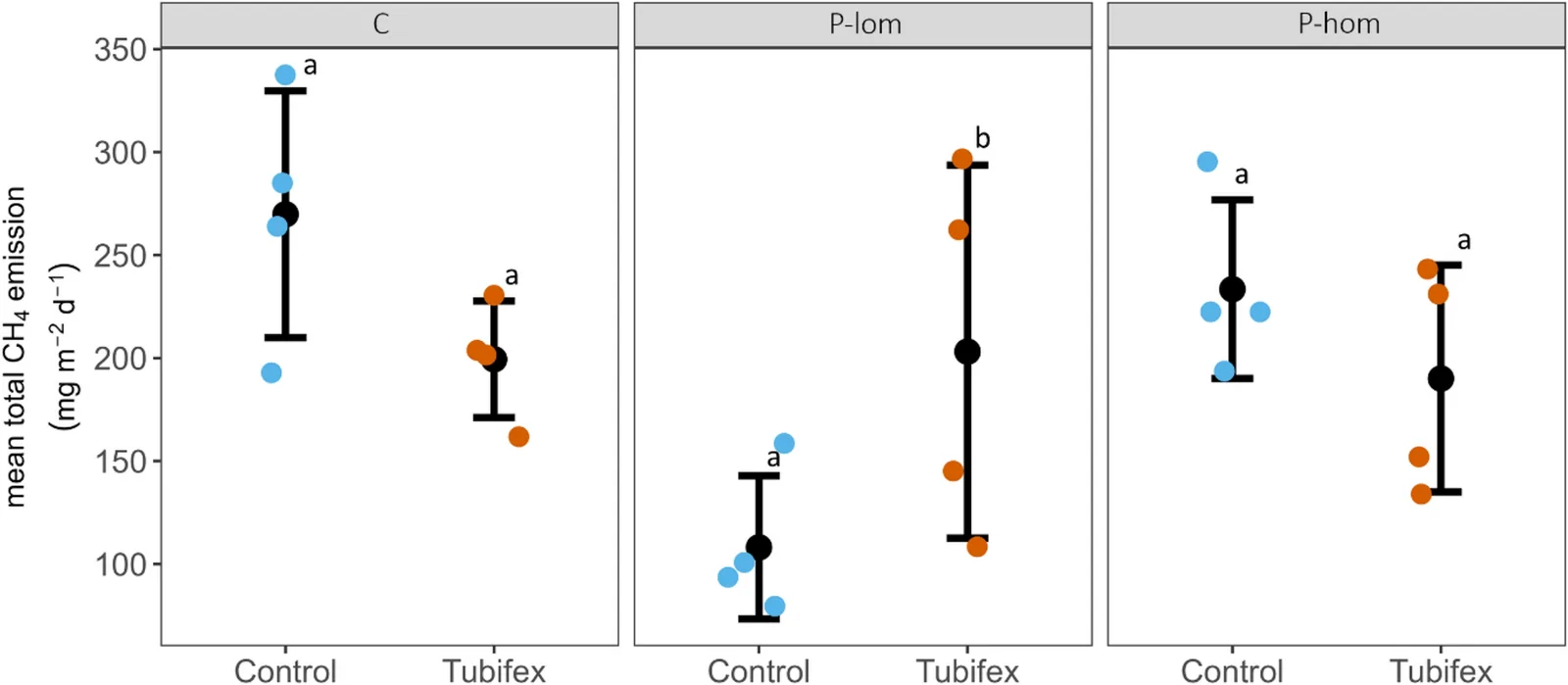
Mean total methane (CH_4_) emissions (mean CH_4_ diffusive + mean CH_4_ ebullition, *n* = 4) with and without *Tubifex* spp. Colour dots indicate the mean values for each microcosm, black dots indicate the mean, blue colour represent “control” and orange colour “*Tubifex* spp.” treatment. The vertical lines represent the standard deviation, and the letters on the top of the bars (**a**, **b**) the pairwise comparison after the LM. Each panel shows a different sediment type: C = clay sediment, P = peat sediment, lom = low organic matter, hom = high organic matter

Total mean CH_4_ emissions (i.e. diffusive + ebullitive) among treatments ranged from 79 to 335 mg m^−2^ d^−1^. The total mean CH_4_ emissions were significantly affected by *Tubifex* presence (LM, F_1,18_ = 5.7, *p* = 0.02), sediment type (LM, F_2,18_ = 9.2, *p* = 0.001) and their interaction (LM, F_2,18_ = 5.0, *p* = 0.01). While total mean CH_4_ emissions were similar between treatments in C and P-hom sediments, *Tubifex* spp. significantly increased emissions in P-lom sediments by 98% (Tukey’s test *p* = 0.02, Fig. 2).

**Fig. 3:**
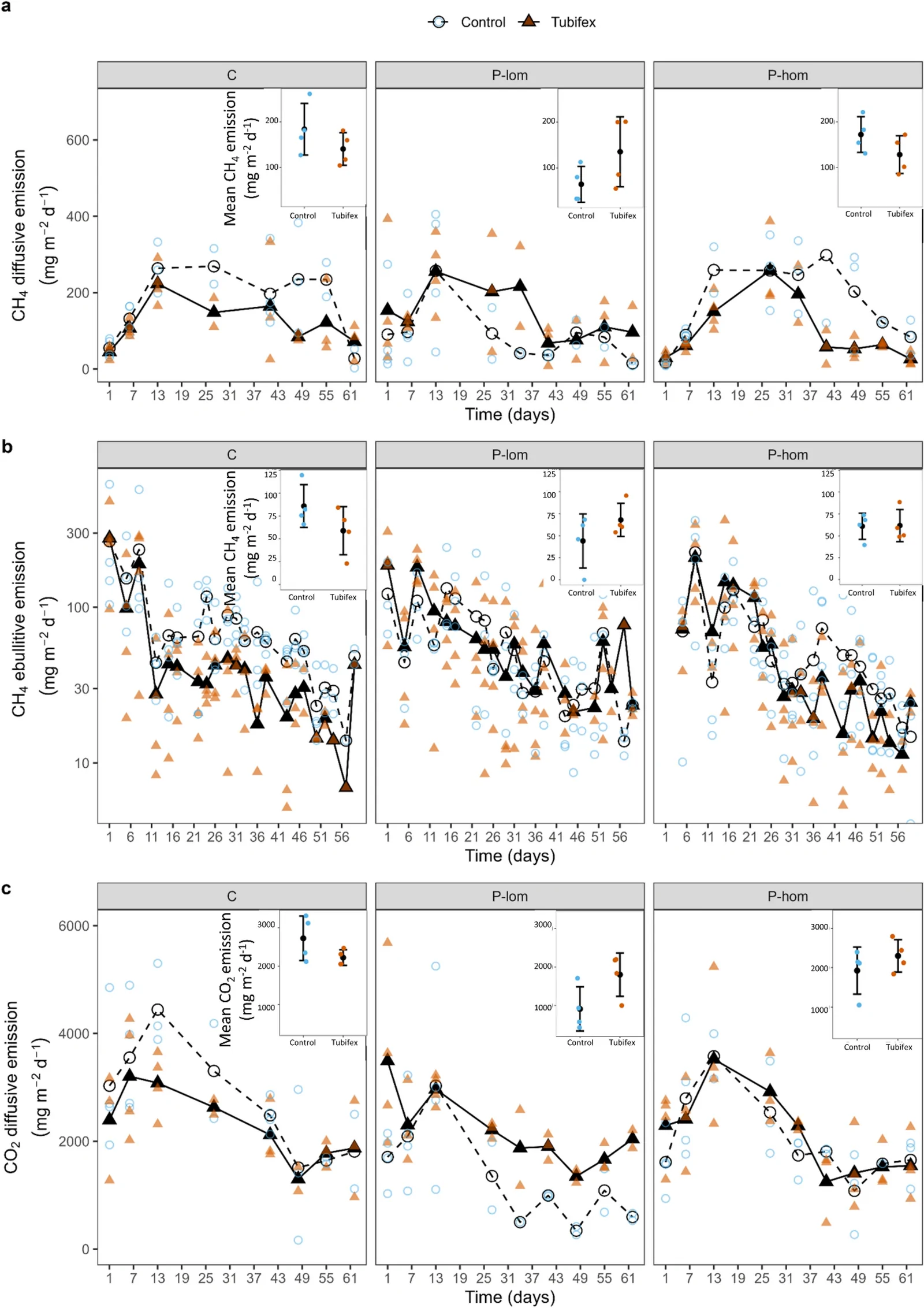
The effect of *Tubifex* spp. on **a** diffusive methane (CH_4_) emissions, **b** ebullitive CH_4_ emissions, and **c** diffusive CO_2_ emissions in three sediments (C = clay sediment, P = peat sediment, lom = low organic matter, hom = high organic matter). The inset figures show cumulative emissions (*n* = 4) expressed as mean daily rates from each greenhouse gas (black dots). Diffusive emissions were integrated over time (area under the curve), and ebullitive fluxes were calculated from collected gas volumes and concentrations normalized by area and deployment time. The orange triangles represent “Tubifex” treatment, and blue circles represent “Control”. Black symbols indicate mean values at a certain timepoint

The diffusive CH_4_ emissions ranged from 2.87 to 476 mg m^−2^ d^−1^ throughout the experimental period. Diffusive CH_4_ emissions from microcosms were significantly influenced by the interaction of *Tubifex* spp. and sediment (LMM, F_2,16.5_ = 2.93, *p* = 0.05). In contrast, no significant effect of sediment, *Tubifex* spp. and experimental time was found (SI, Table S4). Post hoc comparison showed no significant effect of *Tubifex* spp. on diffusive CH_4_ emissions between the sediment types. However, the mean diffusive flux over the entire experimental period tended to be lower when *Tubifex* was present in the P-hom and C sediment (25% and 23%, respectively), while it tended to be higher (110%) in the P-lom sediment (Fig. 3a).

The ebullitive CH_4_ emissions showed high variability among replicates and treatments. They ranged from 5 to 616 mg m^−2^ d^−1^, and showed a strong negative temporal trend (experimental time: LMM, F_1,414.8_ = 223, *p* < 0.001, Fig. 3b). The contribution of CH_4_ emitted through ebullition to the total CH_4_ emissions in our microcosms with *Tubifex* spp. was slightly lower in P-lom (36%) and C (29%) compared to their controls (40% and 32%, respectively). Ebullitive emissions were not significantly affected by *Tubifex* spp., sediment type and their interactions (SI, Table S4). Although not significant, mean CH_4_ ebullition from the C sediment was slightly lower (31%) with *Tubifex* spp. compared to the control.

The diffusive CO_2_ emissions in our microcosms ranged from 165 to 5682 mg m^−2^ d^−1^ throughout the experimental period. Higher CO_2_ emissions were observed during the first five weeks. The diffusive CO_2_ emissions were significantly influenced by *Tubifex* spp. (LMM, F_1,17_ = 3.6, *p* = 0.01), sediment type (LMM, F_2,18_ = 3.0, *p* = 0.006) and was modulated by the experimental time (LMM, F_1,143_ = 57.9, *p* < 0.001). In contrast, mean CO_2_ emissions were significantly affected by the interaction of *Tubifex* spp. and sediment type (LM, F_2,18_ = 3.8, *p* = 0.04). Particularly in P-lom, mean diffusive CO_2_ emissions were 98% higher with *Tubifex* spp. when compared to the control (LM, Tukey’s test *p* = 0.02, Fig. 3c). No significant differences between *Tubifex* spp. and the control were found in C and P-hom sediments.

### Relationship between greenhouse gas (GHG) emissions and benthic biological processes

**Fig. 4:**
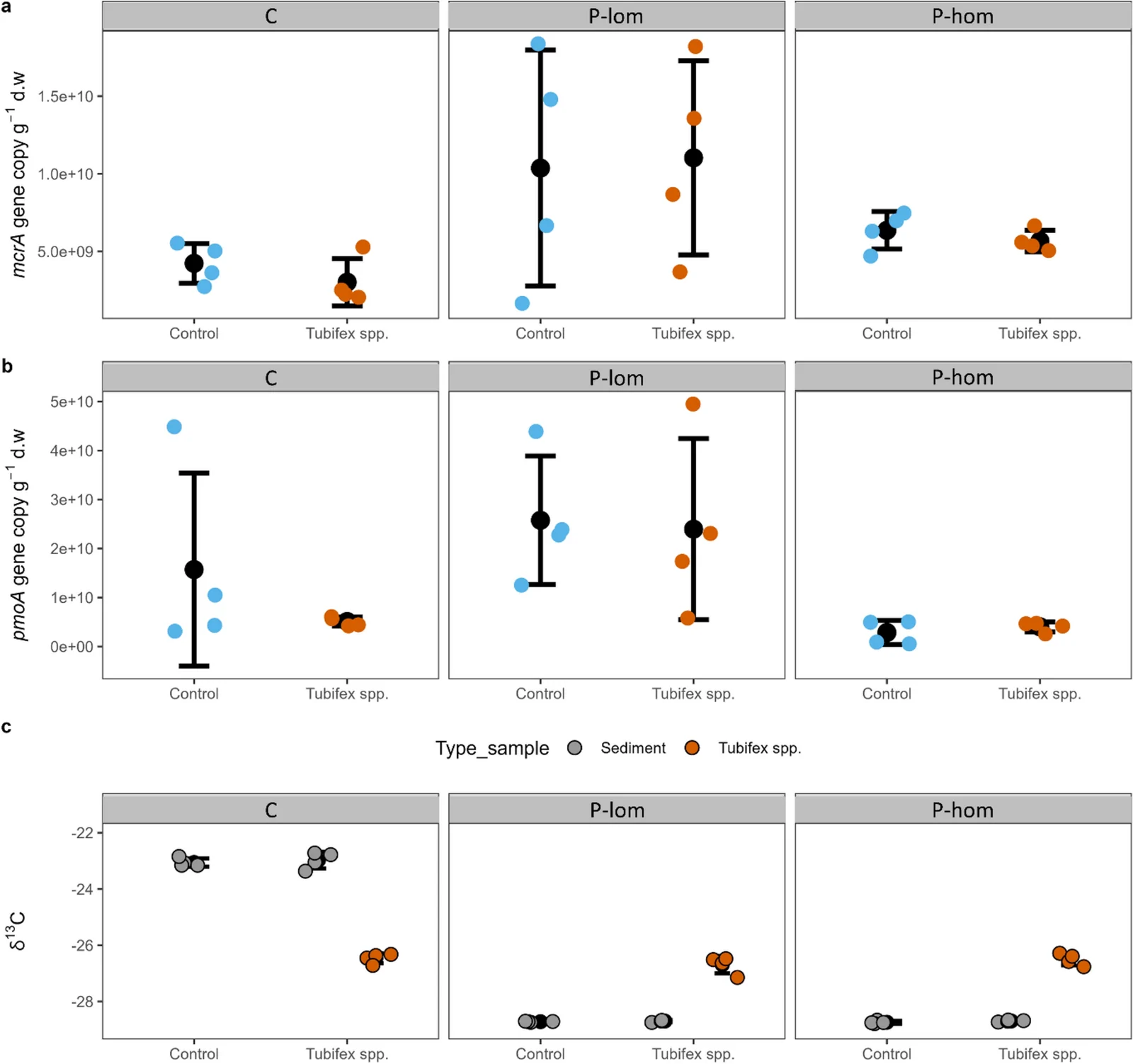
Differences in *mcrA* copy number g^−1^ dw of sediment (**a**), *pmoA* copy number g^−1^ dw of sediment (**b**) and δ^13^C values of sediment and *Tubifex* spp. individuals among sediments with or without *Tubifex* spp (**c**). Grey colour indicates δ^13^C values from sediment samples and orange colour from *Tubifex* samples (**c**). The vertical lines represent the standard deviation and the black dots represent the mean

The abundance of methanogens (gene copy g^−1^ d.w.) did not significantly differ with and without *Tubifex* spp. in all the sediments (Two-Way ANOVA, *p* = 0.42, Fig. 4a). The MOB abundances also did not significantly differ between sediment with *Tubifex* spp. and the controls (Fig. 4b). Sediment type, however, was found to significantly influence methanogenic and MOB abundances (Two-Way ANOVA*, p* = 0.01, *p* < 0.001, respectively).

The δ^13^C of *Tubifex* individuals in all sediments ranged from − 26 to − 27 ‰, while sediment δ^13^C ranged from − 29 to − 23 ‰ (Fig. 4c). The δ^13^C signatures in C sediments were higher than those measured in *Tubifex* individuals. In P-lom and P-hom, the opposite was observed, with sediments showing lower signatures of δ^13^C than in *Tubifex* individuals. The average worm density at the end of the experiment was 1.5 individuals cm⁻^2^.

**Fig. 5:**
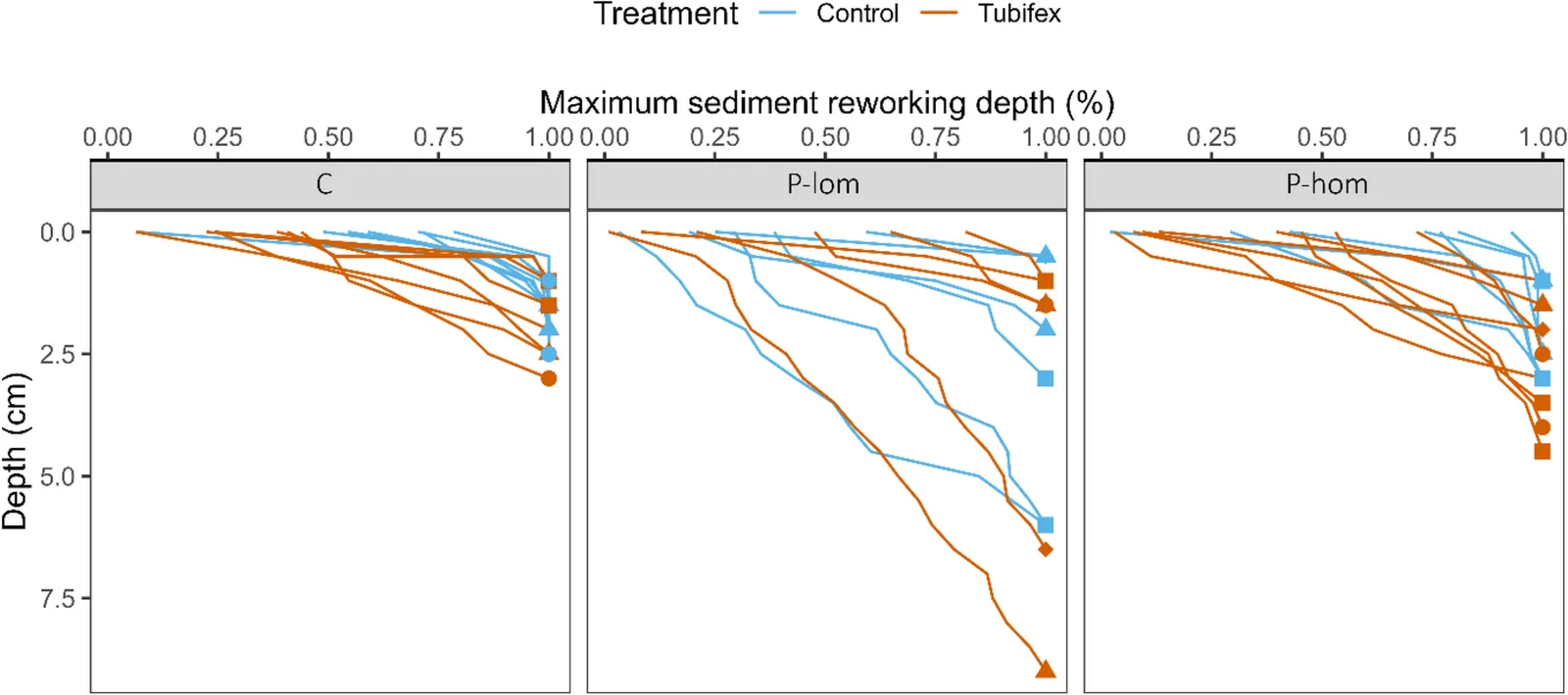
Maximum sediment reworking depth in microcosms with and without *Tubifex* spp. Each line represents the distribution of luminophores normalized by their total number per sampling core. Two cores were extracted per microcosm. Identical symbols per color indicate duplicates. Panels show the three different sediments: C = clay sediment, P = peat sediment, lom = low organic matter, hom = high organic matter

The ΣLUM of all luminophores transported below the sediment water interface varied considerably, with a significant interaction between sediment type and the presence of *Tubifex* spp. (Two-Way ANOVA, *p* = 0.05). *Tubifex* spp. activity increased the ΣLUM by 68% in C and by 113% in P-hom compared to the controls. However, these differences were not significant (Tukey’s test, *p* = 0.73 and *p* = 0.21, respectively). *Tubifex* spp. significantly decreased the ΣLUM by 51% in P-lom sediments (Tukey’s test *p* = 0.03). Conversely, *Tubifex* spp. presence significantly affected the maximum sediment reworking depth (MSRD) values in our microcosms (Two-Way ANOVA, *p* = 0.02, Fig. 5). The MSRD significantly increased by 111% in C and 79% in P-hom in microcosms with *Tubifex* spp., whereas in P-lom it decreased by 7%. In microcosms with *Tubifex* spp. we observed the highest MSRD in P-lom at 2.5 cm, followed by P-hom at 2.3 cm and C at 1.72 cm depth. Methane and CO_2_ emissions were not significantly related to bioturbation intensity parameters (i.e., MSRD and ΣLUM, SI, Table S5) Table 2.

**Table 2:** Overview of the effects of *Tubifex* spp. bioturbation activity on benthic variables and their relationship with methane (CH_4_) and carbon dioxide (CO_2_) emissions

Measurements	*C*	*P-lom*	*P-hom*
CH_4_ diffusion	**0**	** + **	**-**
CH_4_ ebullition	**-**	**0**	**0**
Total CH_4_ emission	**0**	** + **	**0**
CO_2_ diffusion	**0**	** + **	**0**
*mcrA* (CH_4_ producers)	**0**	**0**	**0**
*pmoA* (CH_4_ oxidizers)	**0**	**0**	**0**
O_2_ surface water	**0**	**-**	**-**
MSRD	** + **	**-**	** + **

“- “ indicates a negative effect between variables and *Tubifex* spp. treatment, “ + ” indicates a positive effect between variables and *Tubifex* spp. treatment and “0” indicates no significant effect observed (Tukey’s test). *MSRD* Maximum sediment reworking depth. *C* clay sediment, *P* peat sediment, *lom* low organic matter, *hom* high organic matter.

### Relationship between greenhouse gas (GHG) emissions and biogeochemical properties

According to our PLS analysis, the presence of *Tubifex* spp. primarily altered the direction of correlations between biogeochemical properties and mean CH_4_ emissions (i.e., diffusive and ebullitive), while *Tubifex* presence appeared as an important factor influencing ebullitive CH_4_ and diffusive CO_2_ emissions. In microcosms with *Tubifex* spp. the most significant drivers of mean diffusive CH_4_ emissions (VIP > 1) were porewater PO_4_^3−^​, NH_4_^+^​, NO_3_^−^​, *mcrA* gene abundance and total inorganic carbon (TIC) in surface water (Fig. S4-a), with PO_4_^3−^​, *mcrA* and TIC negatively correlated and NH_4_^+^ and NO_3_^−^ positively correlated with diffusive CH_4_ emissions. In contrast, in microcosms without *Tubifex* spp., diffusive CH_4_ emissions were negatively correlated with NH_4_^+^ and NO_3_^−^, and positively linked to porewater TIC and DOC, as well as *pmoA*-gene copy number in sediment and surface water DOC (Fig. S4-a). Importantly, *Tubifex* presence was highlighted as one of the main drivers of mean ebullitive CH_4_ emissions, together with NH_4_^+^​, surface water TIC, *pmoA-*gene copy number and O_2_ concentrations (69.5%, Fig. S4-c). Although O_2_ concentrations and TIC appeared as important drivers in the control microcosms as well, they showed opposite correlations when compared with microcosms with *Tubifex* spp. While TIC, *Tubifex* presence, and *pmoA* showed a negative correlation, and NH_4_^+^​ and O_2_​ had a positive correlation in microcosms with *Tubifex* spp., this was not the case in the controls. Here, ebullitive CH_4_ emissions were positively explained by *mcrA* gene copy numbers in sediment, and by PO_4_^3−^​​, TIC (surface water) and DOC (surface and porewater), and negatively by O_2_​ in surface water (Fig. S4-d). Lastly, in microcosms with *Tubifex* spp., *Tubifex* presence, *mcrA* abundance, *pmoA* abundance, and NH_4_^+^ (porewater) were significant drivers of CO_2_ emissions (75.5%, Fig. S4-e). In contrast, this was not the case for the controls, where PO_4_^3−^, NO_3_^−^, TIC, and DOC (both surface and porewater) were the most influential drivers of CO_2_ emissions (67.4%, Fig. S4-e).

## Discussion

Our results indicate that the effects of *Tubifex* spp. on CH_4_ and CO_2_ emissions differ between clay (C) and peat (P) sediments, as well as between peat sediments having low (P-lom) and high (P-hom) organic matter content. In two of the three sediments tested (P-hom and C), bioturbation by *Tubifex* spp. was associated with lower CH_4_ emissions. In contrast, *Tubifex* activity in the other sediment (P-lom) significantly increased total mean CH_4_ emissions. The latter was particularly attributed to the increase in diffusive CH_4_ emissions. However, the hypothesized processes underlying these results were not supported as Tubificid-induced changes in MOB abundance, methanogenic abundance, O_2_ penetration depth and bioturbation intensity in sediments could not explain these variations (Fig. 1b). *Tubifex* presence affected CO_2_ emissions, leading to higher mean CO_2_ emissions in one sediment type (P-lom) compared to worm-free microcosms (in line with Fig. 1a). Contrary to our hypothesis (Fig. 1a), Tubifex-induced increases in sediment reworking depth or sediment reworking rates, reflected by higher ΣLUM or MSRD in sediments did not translate into higher CO_2_ emissions. These interpretations should, however, be considered in the context of temporally varying bioturbation intensity rather than a constant *Tubifex*-mediated effect, as *Tubifex* densities declined substantially in our microcosms over the course of the experiment.

Despite the common limitations of experimental systems, in terms of total CH_4_ (mean 108 to 270 mg m^−2^ d^−1^) and CO_2_ (mean 909 to 2726 mg m^−2^ d^−1^) fluxes, the emissions from our microcosms fell within ranges reported for eutrophic freshwater systems (Hendriks et al. 2024). In our microcosms, emissions declined over time, likely reflecting depletion of labile organic matter and resulting from shifts in microbial activity, indicating that bioturbation effects also vary temporally as substrate quality changes. Diffusive and ebullitive CH_4_​ emissions were of similar magnitude (~ 1:1), consistent with field observations available for the peat sediment high in organic matter (Van Der Knaap et al. 2025).

### ***Tubifex*** spp.*** impact on carbon dioxide (CO***_***2***_***) emissions***

Although *Tubifex* spp. presence appeared to be highly influential in explaining the variation in CO_2_ emissions (PLS, Fig. S4-e), we found no consistent *Tubifex-*induced increase in CO_2_ emissions based on the presence/absence analyses. This contrasts with earlier findings (Colina et al. 2021; Mehring et al. 2017). We attribute these contrasting results to the overall high CO_2_ emissions observed in our systems (mean 1800 to 2300 mg m^−2^ d^−1^) combined with the low dissolved O_2_ in the overlying water of our microcosms (Fig. S5). While respiration by tubificid worms contributes directly to CO_2_ production in the systems, their respiration rates decrease drastically under low O_2_ conditions (Lou et al. 2013; Seuβ et al. 1983), which strongly reduces their contribution to the already high CO_2_ production rates. In addition, because the O_2_ concentrations in the water column were low, any Tubifex-mediated O_2_ enhancement in sediments was likely restricted to very localized and short-lived oxic microsites adjacent to burrows (Brand et al. 2013). This interpretation is supported by the occurrence of anoxic conditions below 1 mm sediment depth (Fig. S5). Such dynamics may have resulted in negligible Tubifex-induced effects on CO_2_ production and emissions in our microcosms. Similar to our results, recent studies in sediments characterized by high microbial mineralization rates have reported no significant effects of macrofaunal bioturbation on CO_2_ emissions, despite active sediment reworking (Ganglo et al. 2024; Temmink et al. 2021). However, because we did not measure *Tubifex* respiration rates and O_2_ concentrations inside their burrows, these proposed mechanisms remain inferential.

More broadly, these findings suggest that the effects of tubificid bioturbation strongly depend on the trophic status and O_2_ conditions of the system. In oligotrophic environments, tubificid bioturbation can enhance sediment oxygenation and alter microbial community composition (Mermillod-Blondin et al. 2001; Mermillod‐Blondin et al. 2004; Navel et al. 2012; Saaltink et al. 2019). In contrast, bacterial abundance in eutrophic microcosms containing *Tubifex tubifex* has been shown to vary little relative to controls (Mermillod‐Blondin & Lemoine, 2010). Notably, in the clay treatment, the CO_2_ emissions in the presence of *Tubifex* tended to be lower than in the control. We speculate that this is due to the lower ebullition rates in the *Tubifex* treatment. The higher ebullition rates in the control treatment (Fig. 3b) likely resuspended OM-rich particles. This labile OM was then exposed to O_2_ in the water column, potentially increasing decomposition, thereby increasing CO_2_ production (as reported in studies by Fendinger et al. 1992 and Schwarz et al. 2023). In addition, ebullition-induced resuspension followed by redeposition of suspended sediment could have contributed to the apparent burial of luminophores in the control treatments (Fig. 5). The magnitude of this process is expected to depend on sediment physical properties, including texture and settling behaviour, and therefore may differ among sediment types. This may also explain why variations in carbon emissions were not related to bioturbation intensity proxies in the LM models (SI, Table S5).

### ***Modest impact of Tubifex*** spp.*** on methane (CH***_***4***_***) emissions***

The effects of *Tubifex* spp. on CH_4_ cycling should be interpreted in the context of the persistently low O_2_ conditions observed in our microcosms. Limited O_2_ availability likely constrained the formation and persistence of aerobic microzones within *Tubifex* burrows, thereby reducing the potential for CH_4_ oxidation despite active sediment reworking. This may explain why *Tubifex* spp. did not significantly affect *pmoA* and *mcrA* gene abundances, despite shifting the relationships between carbon emissions and the abundances of methanotrophs and methanogens. Moreover, sediment oxygenation induced by Tubificidae is known to vary with worm density (Saaltink et al. 2019), and the relatively low densities used in our study (~ 1.5–3 ind cm⁻^2^), compared with those reported for similarly eutrophic systems (~ 5.1 ind cm⁻^2^; Mehring et al. 2017), may have further limited shifts in microbial composition and abundance. Nevertheless, porewater CH_4_ concentrations at the end of the experiment were higher in treatments containing *Tubifex* spp. (Fig. S7a), suggesting that sediment reworking redistributed organic matter and stimulated methanogenesis. Increased CH_4_ availability could, in turn, promote localized CH_4_ oxidation where O_2_ remained available (Nijman et al. 2021). These opposing processes, enhanced methanogenesis driven by organic matter redistribution and localized CH_4_ oxidation associated with bioturbation-induced O_2_ transport, may therefore have partially offset one another, as previously reported for other bioturbating invertebrates (Kajan & Frenzel 1999). This balance between competing processes may explain the absence of a consistent net effect of *Tubifex* spp. on CH_4_ emissions despite substantial alterations in sediment functioning. Taken together, the observed patterns are consistent with the mechanisms proposed above; however, the underlying processes were not directly resolved by our experimental approach.

Although *Tubifex* presence was strongly associated with ebullitive CH_4_ emissions (PLS, Fig. S3c), our measurement approach did not detect significant treatment differences. Nevertheless, the observed patterns (Fig. 3) suggest that microcosms containing *Tubifex*, particularly in the C and P-hom sediments, generally exhibited lower ebullitive CH_4_ emissions than the controls. This reduction is likely linked to altered burrowing and reworking activity (supporting hypothesis Fig. 1c) as *Tubifex* significantly increased MSRD in both sediments relative to treatments without bioturbators. Importantly, conclusions regarding ebullition must remain cautious because our ebullition measurements covered only ~ 8.3% of the sediment surface. Given the potentially high spatial heterogeneity of ebullition pathways, this spatial limitation may have reduced our ability to detect treatment effects. Future work would benefit from higher spatial integration of ebullitive fluxes by having multiple bubble traps.

Together, our findings suggest that sediment type, both in terms of sediment classification (mineral—clay versus organic—peat) and OM content, plays a central role in shaping both baseline emissions and the magnitude of *Tubifex*-induced effects. Sediment type was the primary control on GHG emissions, and largely constrained the magnitude and direction of *Tubifex* effects. Dissolved organic carbon and OM are generally expected to enhance microbial mineralization and associated CO_2_​ and CH_4_​ production (Allesson et al. 2020), and together with other sediment properties such as porosity, grain size, and microbial community structure can strongly regulate gas production and transport. Accordingly, *Tubifex* effects were context-dependent and strongly shaped by sediment type. While porewater chemistry (DOC, TIC, NO_3_^−^​, NH_4_^+^​, PO_4_^3−^​) was primarily controlled by sediment type (Figs. S7 and S8), *Tubifex* altered the relationships between these variables and carbon emissions, indicating that bioturbation acted by modifying process linkages rather than baseline solute concentrations. This supports the view that *Tubifex* primarily influences sediment biogeochemistry through localized changes in redox conditions and microbial activity rather than through bulk sediment mixing alone.

## Conclusions

In our two-month microcosm experiment using three different sediment types, we found that the influence of *Tubifex* spp. on CH_4_ and CO_2_ emissions was modulated by sediment type. While *Tubifex* spp. activity increased mean diffusive CO_2_ and total CH_4_ emissions by, respectively, 98% and 88% in peat sediments, low in organic matter (P-lom), no or only weak effects were observed in peat sediments, high in organic matter (P-hom), and clay sediments (C). Moreover, the mechanisms driving these changes—or their absence—may be linked to how sediment properties modulate *Tubifex* bioturbation activities (e.g., reworking intensity) and alter the relationship among oxygen, organic carbon, microbial abundance (i.e., methanogens and methanotrophs), nutrient availability and gaseous carbon emissions. For ebullitive CH_4_ emissions, we did not detect a significant effect of *Tubifex*. However, these results should be interpreted in the context of our measurement approach and the decline in *Tubifex* densities over the course of the experiment, which reduced bioturbation intensity over time. Our findings indicate that although predicting site-specific effects of bioturbators remains challenging, they can influence carbon emissions. Moreover, the variation in bioturbator presence and abundance may help explain a portion of the previously unexplained variance in CO_2_ and CH_4_ emissions observed in field studies (e.g., Hendriks et al. 2024). However, better context-dependent insight into their quantitative effects on GHG emissions is needed to incorporate their presence when predicting carbon emissions. Further studies should aim to more precisely disentangle the mechanisms through which bioturbators and sediment characteristics interact to shift biogeochemical processes and bioturbation activities, resulting in changes in GHG emissions.

## Supplementary Information

Below is the link to the electronic supplementary material.

Supplementary file1 (DOCX 1761 KB)

## Data Availability

The datasets generated during and/or analysed during the current study are available in the DANS-EASY online repository under: https://doi.org/10.17026/LS/5O6HBM.
