# Supplementary material for: Sediment properties modulate the bioturbation effects of Tubifex spp. on CH4 and CO2 emissions in freshwater ecosystems
Source: Biogeochemistry. 2026 Aug 19;169(6):72. doi: 10.1007/s10533-026-01364-6 (PMC13768370; doi:10.1007/s10533-026-01364-6)
Supplement: Supplementary file 1 [file 10533_2026_1364_MOESM1_ESM.docx]

*Biogeochemistry*

**Sediment properties modulate the bioturbation effects of *Tubifex* spp. on CH_4_ and CO_2_ emissions in freshwater ecosystems**

Ilenia Marquina-Luévano^1^, Lucía Cabrera-Lamanna^1^, Annelies J. Veraart^1^, Andreas Lorke^2^, Dick van Oevelen^3^ , Sarian Kosten^1^

^1^ Department of Ecology, Radboud Institute for Biological and Environmental Sciences, Radboud University, Nijmegen, the Netherlands.

^2^ Institute for Environmental Sciences, University of Kaiserslautern-Landau (RPTU), Landau, Germany.

^3^ Royal Netherlands Institute for Sea Research – NIOZ-Yerseke, the Netherlands.

**Corresponding author:**

Ilenia Marquina-Luévano; Email: ilenia.marquinaluevano@ru.nl

**Supplementary Information**

This Supplementary Information (SI) provides detailed information on the methodology used to build our microcosms, as well as on the analyses of methane (CH_4_) and other physicochemical parameters (i.e., oxygen, sediment reworking and microbial abundance). This SI provides detailed data on the protocols used to create artificial water and sediments (Table S1 and S2), a schematic of our experimental setup (SI- Fig.1) and how we estimated the mean CH_4_ and CO_2_ emissions and the CH_4_ lost from our microcosms through the outflow. It also includes more detailed information on the methodology used to quantify CH_4_ concentrations in water, as well as oxygen (O_2_), luminophores and microbial abundances in the sediment. The SI also includes figures that illustrate the effects of *Tubifex* spp. on total CH_4_ emissions, nutrients and their relationship with physicochemical parameters. Lastly, the SI also includes a summary of the results of the statistical analysis used to explain the effects of our treatment and bioturbation intensity on greenhouse gas (GHG) (Table S4 and Table S5, respectively).

*Supplementary methods*

M1: Artificial rainwater

**Table S1.** Artificial rainwater composition. Rainwater constituents were dissolved in deionized water, after which the prepared solution was stored at room temperature (25 °C) until setting up the microcosms.

| *Chemical components* | *Concentration*  *(µmol/L)* | *Molecular weight (g/mol)* | *Concentration*  *(g/L)* |
| --- | --- | --- | --- |
| *NaCl (food-grade sea salt)* | *-* | *-* | *5.00E-03* |
| *KCl* | *30* | *74.55* | *2.24E-03* |
| *CaCl_2_ · 2H_2_O* | *10.2* | *147.02* | *1.50E-03* |
| *Fe-EDTA* | *10.1* | *345.06* | *3.49E-03* |
| *KH_2_PO_4_* | *1* | *136.09* | *1.36E-04* |
| *ZNSO_4_ · 7H_2_O* | *0.7* | *287.54* | *2.01E-04* |
| *MnCl_2_ · 4H_2_O* | *0.8* | *197.91* | *1.58E-04* |
| *CuSO_4_ · 5H_2_O* | *0.2* | *249.69* | *4.99E-05* |
| *H_3_BO_3_* | *0.8* | *61.83* | *4.95E-05* |
| *(NH_4_)_6_Mo_7_O_24_ · 4H_2_O* | *0.008* | *1236.01* | *9.89E-06* |

M2: Artificial sediment

**Table S2.** Artificial sediment was constructed using silver sand, mineral clay and 27% of grained particles from dry plants as ‘fresh’ organic matter input (OM, Table S2). All components were obtained commercially in the Netherlands. Deionized water (DI water) was added to all dry components and manually mixed to add moisture. This sediment was further used to measure sediment reworking depth.

| *Sediment components* | *Weight*  *(Kg)* |
| --- | --- |
| *Silver sand* | 1.200 |
| *Mineral clay* | 1.200 |
| *Dry plants* | 0.648 |
| *DI water* | 3.400 |

M3: Experimental set-up

**Supplementary Figure 1.**  Experimental design using microcosms. “a” Represents control treatments, “water dripping” of low-oxygenated water and how bubble traps were placed in each mesocosm. “b” Represents treatments with *Tubifex* spp. and the acrylic lid, which was connected to a greenhouse gas (GHG) analyzer. “c” represents the compartment where O_2_ measurements were performed. OM = Organic matter.

The aerated water was prepared in separate reservoirs supplied with a constant flow of ambient air (21% O₂). Artificial rainwater was delivered to each microcosm via silicone tubing (Ø 7.9 mm) connected to a Masterflex L/S peristaltic pump (Model 7528–30, Masterflex LLC, USA) and added as droplets (at approximately 0.3 L^-1^ d^-1^ rate) throughout the duration of the experiment. Each microcosm was equipped with a small overflow outlet to maintain a constant water level. Methane loss via the overflow accounted for only a minor fraction (5–6%) of total CH_4_ emissions. We estimated the CH_4_ lost from the microcosms (aCH_4_ in mg/m^2^/d) through the outflow by multiplying the water flow rate $(Q)$ by the CH_4_ concentrations in the surface water (_c_CH_4_) and divided by the surface area (A). Next, we expressed this loss as a percentage of the total CH_4_ gaseous emissions from each mesocosm.

$$a{CH}_{4}=\frac{Q \times c{CH}_{4}}{A}$$

(eqs. S1)

M4: *Tubifex* spp. count for mortality

At the end of the experiment, we homogenized the sediment and collected one sediment core (radius 1.5 cm, height 3.2 cm; volume ≈ 22.6 cm³) from each microcosm. A 20 mL subsample of sediment was used to quantify *Tubifex* spp. abundance. The number of individuals counted in the subsample was first scaled to the total core volume and subsequently expressed per unit surface area by dividing by the core cross-sectional area (7.07 cm²). These yielded areal densities in individuals per cm⁻². Calculations assume a uniform distribution of organisms within the sampled sediment layer.

M5: Methane concentrations in water

Surface water samples for CH_4_ analyses were taken at the beginning and end of the experiment by submerging a syringe connected to a pre-vacuumed 12 mL exetainer 2 cm below the water surface, allowing the exetainer to fill with surface water. For porewater samples, we used a ceramic Rhizon sampler (pore size 0.12/0.18 μm, Rhizon SMS 10 cm, Rhizosphere Research, the Netherlands) permanently placed at 5 cm depth into the sediment of each aquarium, which we connected to a vacuumed exetainer. Each exetainer was previously prepared with 0.1 mL of 2.5 M H_2_SO_4_ to slow down microbial processes. In the exetainers full of water, we created a headspace by adding N_2_ gas and allowing the same volume of water to escape through a syringe connected to the septum. In exetainers that were not totally full (e.g., due to clogging of the rhizon), we equilibrated the headspace to neutral pressure by adding N_2_. Before measurements, all exetainers were vigorously shaken 10 times to allow equilibrium between gas concentrations in water and headspace. The concentration of dissolved CH_4_ in water was calculated using Henry’s law and considering the CH_4_ solubility constant and temperature conditions (Sander, 2015).

M6: Oxygen in sediment

At the end of the experiment, we measured dissolved O_2_ profiles in the sediments to assess whether the presence of Tubifex spp. influenced oxygen distribution. Measurements were taken at depths from 0 to –10 mm below the sediment–water interface using a Microx TX3 oxygen microsensor with a flat-broken needle tip (Presens, Regensburg, Germany), in one replicate per treatment (SI, Fig. 1-c).

M7: Quantification of sediment reworking depth

The camera was set to have ISO: 400, AV: 5.6, TV: 1/10, and manual focus. Before taking pictures we set up the camera and the sediment core in separate stable tripods to prevent movement and distance bias in our analysis. Images were originally saved in C2 format and later transformed into TIFF to preserve the image quality. The macro script opens each image and converts it to an RGB stack. First, we defined the threshold to distinguish luminophore particles from sediment (53= sediment, 255= luminophores) with a black background (Fig.1-A). We selected the quantification area for luminophores using an oval shape figure. Next, we ran the tool ‘Analyze particles’ with specifications of size and circularity that detect only luminophores. Ultimately, we received the number of luminophores detected in each image using the function ‘display clear summarize add slice’ (Fig.1-B,C). This data was immediately recorded in an Excel data sheet.

**Supplementary Figure 2.** Luminophore particle analysis, “a” is the original image in TIFF format, “b” shows the threshold used to detect particles and “c” shows the particles counted in ImageJ.

M8: Methanotrophic and methanogenic abundance

Reagents used in qPCR were: 2X SensiFAST SYBR green (BIOLENE, 10 µl), elution buffer (MACHEREY-NAGEL, 0.8 µl), forward primer (0.6 µl), reverse primer (0.6 µl) and sample DNA (2 µl). The qPCR followed the following thermal cycle: An initial cycle of 3 minutes denaturation at 95°C, 45 cycles of 10s at 95°C, 15s at 58°C, 25s at 72°C and 10s at 82°C. An increase of 1°C from 70°C to 95°C was used for the last melt-curve of each run (Costello & Lidstrom, 1999).

**Table S3.** The primers combinations and primer sequences used in qPCR assays of *mcrA* gene abundance, as a proxy for methanogens, and *pmoA* gene abundance as a proxy of methanotroph abundance.

| *Gene* | *Primer sets* | *Primer sequence (5’-3’)* | *Reference* |
| --- | --- | --- | --- |
| *mcrA* | mlas  mcrA-rev | *GGTGGTGTMGGDTTCACMCARTA*  *CGTTCATBGCGTAGTTVGGRTAGT* | (Steinberg & Regan, 2008) |
| *pmoA* | A189F  Mmb661R | *GGNGACTGGGACTTCTGG*  *CCGGMGCAACGTCYTTACC* | (Costello & Lidstrom, 1999) |

M9: Mean emissions of methane (CH_4_) and carbon dioxide (CO_2_)

For mean diffusive emissions, we first calculated total emission as the area under the curve (AUC) of flux rates (determined every 7 days) versus time plots using a numerical integration with the trapezoidal rule. Mean emissions were later calculated by dividing total emissions by the total experimental time (62 days). Mean CH_4_ ebullitive emissions were calculated by multiplying the collected volumes ($V_{ebb}$) by the respective CH_4_ concentrations ($p{CH}_{4 gas}$) and dividing by the funnel area ($A$ = 0.003 m^2^) and deployment time (t= 50-52 days).

$$\varphi eb= \frac{\Sigma(p{CH}_{4 gas}X V_{ebb})}{t*A}$$

(eqs. S2)

For the dates $p{CH}_{4 gas}$ no data were available, $p{CH}_{4 gas}$ was estimate based on the average of the temporally closest CH_4_ concentration measurement. To visualise total CH_4_ emissions per date of measurement, we summed diffusive and ebullitive emissions per replicate at each sampling point. The mean values were then later calculated as the average across replicates.

*Supplementary figures*

**Supplementary Figure 3.** Partial Least Squares (PLS) loading plots showing the relationship between mean methane (CH_4_) diffusion (with *Tubifex* spp. in “a”, without in “b”), mean CH_4_ ebullition (with *Tubifex* spp. in “c”, without in “d”), and mean carbon dioxide (CO_2_) diffusion (with *Tubifex* spp. in “e”, without in “f”), to various benthic, and pelagic variables. Orange dots show high influential predictors (VIP > 1), blue dots show moderately influential predictors (0.8 < VIP < 1), and grey dots show less influential predictors (VIP < 0.8). Non-normal parameters were log transformed (“log_” as a prefix). Benthic explanatory variables: % of organic matter (OM_sed), δ^13^C of sediment (C13_sed), the concentration in porewater of ammonium, nitrate and phosphate (NH4_pw, NO3_pw and PO4_pw respectively, in mg L^-1^), total inorganic carbon (TIC_pw) and dissolved organic carbon in porewater (DOC_pw in mg L^-1^). Pelagic explanatory variables: O_2_ concentration in surface water (O2_sw in mg L^-1^), total inorganic carbon in surface water (TIC_sw) and dissolved organic carbon in surface water (DOC_sw in mg L^-1^).

**Supplementary Figure 4.** Oxygen concentration in surface water. Triangles represent “*Tubifex*” treatment, and circles represent “Control”. Black figures indicate mean values (as the average of experimental replicates) corresponding to one specific sampling time. Orange triangles and blue circles represent individual measurements from “*Tubifex*” and “Control” treatments, respectively.

**Supplementary Figure 5.** Oxygen profile above and below the sediment-water interface (SWI) in one replicate per treatment. Triangles represent “*Tubifex*” treatment, and circles represent “Control”.

**Supplementary Figure 6.** Sediment porosity from all microcosms. The boxes represent the interquartile range (IQR), spanning from the first to the third quartile. The line inside each box indicates the median, while the whiskers extend to the most extreme values within 1.5 times the IQR from the quartiles.

**Supplementary Figure 7.** Differences in (a) methane (CH_4_) porewater concentrations (b) DOC in surface water and (c) TIC in porewater at the end of the experimental period among sediments with or without *Tubifex* spp. The boxes represent the interquartile range (IQR), spanning from the first to the third quartile. The line inside each box indicates the median, while the whiskers extend to the most extreme values within 1.5 times the IQR from the quartiles.

**Supplementary Figure 8.** Differences in concentrations of (a) nitrate (NO_3_^-^), (b) ammonium (NH_4_^+^) and (c) phosphate (PO_4_^3-^) in porewater at the end of the experimental period among sediments with or without *Tubifex* spp. The boxes represent the interquartile range (IQR), spanning from the first to the third quartile. The line inside each box indicates the median, while the whiskers extend to the most extreme values within 1.5 times the IQR from the quartiles.

*Supplementary tables*

**Table S4.** Summary of LMM models explaining greenhouse gas (GHG) fluxes over time with and without *Tubifex* spp. and LM models for the mean GHG fluxes. Tukey p-values from pairwise comparisons were included when applicable (* for *p*- value ≥ 0.01 and <0.05, ** for *p*-value < 0.01).

| GHG  (mg m^-2^ day^-1^) | Significant *p-value* = *  (ANOVA, Chi2 test) | Significant variables  (pairwise comparisons) | Tukey *p-values* |
| --- | --- | --- | --- |
| CH_4_ diffusion | Treatment x Sediment * | - | - |
| CH_4_ ebullition | Experimental time** | - | - |
| CO_2_ diffusion | Treatment *  Sediment **  Experimental time** | - | - |
| Mean CH_4_ diffusion | Sediment *  Treatment x Sediment * | - | - |
| Mean CH_4_ ebullition | Sediment* | - | - |
| Total mean CH_4_ | Treatment *  Sediment *  Treatment x Sediment * | P-lom x Treatment | 0.02 |
| Mean CO_2_ diffusion | Treatment *  Sediment **  Treatment x Sediment * | P-lom x Treatment | 0.02 |

**Table S5.** Summary of LM models explaining the relationship between mean greenhouse gas (GHG) fluxes and bioturbation intensity (i.e., sum of all luminophores transported into the sediment profile [ΣLUM] and the maximum sediment reworking depth [MSRD]) with and without *Tubifex* spp.

| MSRD model | | | | |
| --- | --- | --- | --- | --- |
| GHG  (mg m^-2^ day^-1^) | Significant predictor  (ANOVA, Chi2 test) | df | F | *P* value |
| Mean CH_4_ diffusion | Sediment | 2,16 | 4.8 | 0.02 |
| Mean CH_4_ ebullition | - | - | - | - |
| Total mean CH_4_ | Sediment | 2,16 | 5.4 | 0.01 |
| Mean CO_2_ diffusion | Sediment  Treatment | 2,16  2,16 | 9.3  9.5 | 0.002  0.03 |
| ΣLUM model | | | | |
| Mean CH_4_ diffusion | Sediment | 2,16 | 4.4 | 0.02 |
| Mean CH_4_ ebullition | - | - | - | - |
| Total mean CH_4_ | Sediment | 2,16 | 5.3 | 0.01 |
| Mean CO_2_ diffusion | Sediment | 2,16 | 9.5 | 0.001 |

**References**

Costello, A. M., & Lidstrom, M. E. (1999). Molecular Characterization of Functional and Phylogenetic Genes from Natural Populations of Methanotrophs in Lake Sediments. *Applied and Environmental Microbiology*, *65*(11), 5066–5074. https://doi.org/10.1128/AEM.65.11.5066-5074.1999

Sander, R. (2015). Compilation of Henry’s law constants (version 4.0) for water as solvent. *Atmospheric Chemistry and Physics*, *15*(8), 4399–4981. https://doi.org/10.5194/acp-15-4399-2015

Steinberg, L. M., & Regan, J. M. (2008). Phylogenetic Comparison of the Methanogenic Communities from an Acidic, Oligotrophic Fen and an Anaerobic Digester Treating Municipal Wastewater Sludge. *Applied and Environmental Microbiology*, *74*(21), 6663–6671. https://doi.org/10.1128/AEM.00553-08
